# Analysis of serum metabolomics in patients with different types of chronic heart failure

**DOI:** 10.1515/med-2025-1210

**Published:** 2025-07-23

**Authors:** Bingzhang Jie, Qiang Li, Ling Han, Liwei Chen, Ming Yang

**Affiliations:** Department of Cardiology, Fuxing Hospital, Capital Medical University, Beijing, 100038, China; Department of Pharmacology, Beijing Laboratory for Biomedical Detection Technology and Instrument, School of Basic Medical Sciences, Capital Medical University, Beijing, 100069, China

**Keywords:** heart failure, LVEF, liquid phase high-resolution mass spectrometry, glycerophospholipid metabolic pathway, metabolome

## Abstract

**Background:**

Heart failure remains a major public health issue, and there are still no reliable biomarkers for left ventricular ejection fraction (LVEF).

**Objective:**

To screen for differential metabolites in the blood of HFpEF, HFmrEF, and HFrEF patients based on metabolomics analysis of their blood samples.

**Methods:**

Total 44 patients in HFpEF group, 30 patients in HFmrEF group, and 36 patients in HFrEF group were selected. The blood metabolites were analyzed by liquid chromatography high-resolution mass spectrometry and classified by principal component analysis, and then potential biomarker were screened. Partial least squares discriminant analysis was used to model and investigate the predictive ability of biomarkers for LVEF.

**Results:**

Blood metabolite profiles of HFpEF, HFmrEF, and HFrEF groups could be well distinguished, and seven potential biomarkers were identified, such as phosphatidylcholine, phosphatidylinositol, lysophosphatidylcholine, lysophosphatidylcholine, ceramide, sphingosine, and sphingomyelin. Four metabolic pathways, such as glycerol phospholipid metabolic pathway, linoleic acid metabolic pathway, purine pyrimidine metabolism pathway, and linolenic acid metabolism pathway were identified, among which glycerol phospholipid metabolism pathway was the most significant.

**Conclusion:**

The changes in glycerol phospholipid metabolism pathway may help identify HFpEF, HFmrEF, and HFrEF.

## 1 Introduction

Heart failure (HF) is a complex syndrome that is the final stage of various cardiovascular diseases. According to left ventricular ejection fraction (LVEF), HF can be divided into HF with preserved ejection fraction (HFpEF, LVEF ≥ 50%), HF with mildly reduced ejection fraction (HFmrEF, LVEF 41–49%), and HF with reduced ejection fraction (HFrEF, LVEF ≤ 40%). Despite significant progress in the diagnosis and treatment of HF, patients still have severe prognosis. Therefore, there is an urgent need to explore new biomarkers for HF.

The occurrence and development of HF involve multiple pathophysiological pathways such as metabolism, neurohormones, and the immune system. At present, many biomarker related to HF have been identified, such as brain natriuretic peptide (BNP), tumor factor TNF, interleukins IL-1β, IL-6, IL-18, IL-33, and Stromelysin-2 [1,2]. However, no laboratory indicators related to LVEF have been found. While BNP may be elevated in HF patients with preserved LVEF, it cannot distinguish HFpEF, HFmrEF, and HFrEF patients. The metabolic disorders in patients with HF are significant, and metabolomics help us understand the metabolic disorders associated with myocardial dysfunction.

Metabolomics refers to the use of systematic methods such as nuclear magnetic resonance, high-performance liquid chromatography, gas chromatography, and mass spectrometry to analyze the changes in endogenous metabolic product profiles in biological fluids and tissues caused by pathological physiological stimuli and genetic modifications. Among them, liquid chromatography-mass spectrometry technology has higher sensitivity and resolution, making it suitable for metabolomics research [3]. Despite many previous studies on the metabolomics of HF, we have very little knowledge on the differences in metabolomics among the patients with HFpEF, HFmrEF, and HFrEF.

Therefore, in this study we aimed to investigate characteristic metabolic spectrum of HF. We employed liquid chromatography-high-resolution mass spectrometry to obtain metabolite profiles of blood samples from patients with HFpEF, HFmrEF, and HFrEF, and conducted bioinformatics analysis of differential metabolites. Representative biomarkers were screened to provide new diagnostic strategies for HF.

## 2 Methods

### 2.1 Instruments and reagents

Following instruments and reagents were used in this study: Waters liquid chromatograph (Watsch, USA); Waters SYNAPT G2-Si mass spectrometer (Watsch, USA); high-speed freezing centrifuge (Sigma, USA), vacuum concentrator (Labconco, USA); methanol, water, acetonitrile, formic acid, and ammonium formate (mass spectrometry pure, Thermo Fisher, USA); and HSS T3 column (2.1 × 100 mm, 1.8 µm) (Watsch, USA).

### 2.2 Subjects

Blood samples were collected from HF patients at Fuxing Hospital affiliated with Capital Medical University, who were selected from 110 patients with congestive HF admitted to the Cardiovascular Department from September 2018 to December 2020. Inclusion criteria included: (1) all met the diagnostic criteria for central HF in the 2018 Chinese Heart Failure Diagnosis and Treatment Guidelines. (2) According to the 2021 ESC guidelines, HF was divided into HF with preserved ejection fraction (HFpEF, LVEF ≥ 50%), HF with mildly reduced ejection fraction (HFmrEF, LVEF 41%–49%), and HF with reduced ejection fraction (HFrEF, LVEF ≤ 40%), with 44 cases in the HFpEF group, 30 cases in the HFmrEF group, and 36 cases in the HFrEF group.

Exclusion criteria were (1) patients admitted without the main cause of HF; (2) those who die in the hospital; (3) patients with chronic HF caused by other reasons (such as blood system, chronic anemic heart disease, endocrine and metabolic factors such as hyperthyroidism heart disease), except for coronary heart disease, high heart disease, and dilated heart disease; (4) congenital heart disease patients with severe complications that require surgical treatment (such as valve disease); (5) patients with liver and kidney failure; (6) those who suffer from other serious systemic diseases such as malignant tumors, hemorrhagic diseases, severe acute infections or metabolic disorders; and (7) those who cannot clearly and accurately describe their medical history due to consciousness disorders, intellectual disabilities, or vague language. The general information of the samples is shown in Table 1. Blood samples were collected immediately upon admission, and centrifuged at 3,000 rpm for 10 min at 4°C. The supernatant was stored at −80°C for future use.

**Table 1:** Baseline characteristics of study patients in temperature groups

Characteristic	LVEF ≥ 50%	LVEF 41–49%	LVEF ≤ 40%	*p-*value
Number (*n*)	44	30	36	
Age (years)	81.3 ± 9.9	77.0 ± 11.8	74.4 ± 15.6	0.100
Gender (male)	18 (40.9%)	18 (60.0%)	24 (66.7%)	0.055
Weight (kg)	65.9 ± 8.7	66.1 ± 9.3	67.7 ± 10.8	0.678
BMI (kg/m^2^)	24.5 ± 2.7	23.8 ± 2.8	24.1 ± 2.8	0.199
Heart rate (beats/min)	81.5 ± 17.1	81.7 ± 18.0	88.6 ± 22.1	0.130
Systolic pressure (mmHg)	140.3 ± 28.4	144.4 ± 24.2	131 ± 24.2	0.100
LVEF (%)	57.4 ± 3.9	45.5 ± 2.5	31.1 ± 5.9	<0.001
LVIDd (mm)	45.0 ± 6.3	50.3 ± 7.1	56.5 ± 8.2	<0.001
TC (mmol/L)	4.6 ± 0.7	4.5 ± 0.8	4.2 ± 0.5	0.873
TG (mmol/L)	1.88 ± 0.57	1.76 ± 0.65	1.54 ± 0.71	0.579
LDL-C (mmol/L)	2.89 ± 0.97	2.78 ± 0.85	2.54 ± 0.88	0.355
HDL (mmol/L)	1.36 ± 0.29	1.27 ± 0.38	1.14 ± 0.42	0.483
BNP (pg/mL)	1432.9 ± 1541.2	1963.5 ± 1468.8	2751.5 ± 1645.3	<0.001
Serum potassium (mmol/L)	4.0 ± 0.9	3.5 ± 0.7	3.7 ± 0.8	0.009
Serum sodium (mmol/L)	133.8 ± 6.1	133.2 ± 5.4	135.1 ± 6.9	0.329
BUN (mg/dL)	39.3 ± 22.5	36.7 ± 20.9	40.3 ± 25.7	0.835
Blood creatinine (μmol/L)	146.4 ± 81.3	162.7 ± 179.9	141.2 ± 75.8	0.975
eGFR (mL/min·1.73 m^2^)	44.6 ± 23.7	50.9 ± 29.6	50.6 ± 25.2	0.548
Blood glucose (mmol/L)	6.8 ± 0.9	6.5 ± 0.7	6.3 ± 0.7	0.729
**Combined diseases**				
Coronary heart disease	37 (84.1%)	28 (93.3%)	33 (91.7%)	0.380
Hypertension	34 (77.3%)	22 (73.3%)	26 (72.2%)	0.862
Diabetes	17 (38.6%)	10 (33.3%)	11 (30.6%)	0.741
Hyperlipidemia	30 (68.2%)	19 (63.3%)	26 (72.2%)	0.742
Atrial fibrillation/atrial flutter	26 (59.1%)	15 (50.0%)	13 (36.1%)	0.123
Valvular disease	12 (27.3%)	9 (30.0%)	9 (25.0%)	0.902
**Medications**				
β Receptor blockers	31 (70.5%)	23 (76.7%)	30 (83.3%)	0.402
ACEI/ARBs	22 (50.0%)	18 (60.0%)	16 (44.4%)	0.447
Spironolactone	16 (36.4%)	20 (66.7%)	25 (69.4%)	0.004
Digoxin	6 (13.6%)	2 (6.7%)	9 (25.0%)	0.111
Loop diuretic	28 (63.6%)	24 (80.0%)	32 (88.9%)	0.026

### 2.3 Sample processing

The samples were thawed at room temperature, and mixed with 400 μL pre-cooled mass spectrometry grade methanol solution (final concentration 80%), fully vortexed, and put on ice for 30 min. After centrifugation at 13,000 rpm for 15 min at 4°C, the supernatants (approximately 200 μL) were collected and centrifuged again at 13,000 rpm for 15 min. The supernatants were subjected to metabolomics analysis.

### 2.4 Metabolomics analysis

Chromatographic column: Waters Acquisition HSS T3 (2.1 × 100 mm, 1.8 µm), injection volume: 5 µL, flow rate: 0.4 mL/min, and column temperature: 40°C. Mass spectrometry conditions: capillary 1.5 kV, sample cone 30 V, desolvation gas temperature 500°C, desolvation gas flow 800 L/h, source temperature 120°C, and mobility carrier gas 32 mL/min. The ion scanning range 50–1,000*m*/*z*, and scanning time 0.2 s.

### 2.5 Data processing

Mass spectrometry contour map was imported into the Waters Nonlinear Progenesis (QI) software, with QC sample contour map as a reference sample alternative. The samples were divided into three groups: normal ejection fraction group, ejection fraction greater than 40% group, and ejection fraction less than 40% group. Principal component analysis (PCA) of unsupervised pattern recognition and partial least squares analysis (PLS-DA) of supervised pattern recognition were performed. Variable importance in the project was calculated based on the PLS-DA model. Differential compounds were identified based on *p*-value < 0.05 and fold change (Max/Min) > 1.2 values, and analyzed with Human Metabolome Database and Kyoto Encyclopedia of Genes and Genomes database. The differential metabolites were imported into MetaboAnalyst 4.0 (www.metaboanalysts.ca) for enrichment analysis, metabolic pathway analysis, and other related bioinformatics analysis.


**Informed consent:** All patients provided written informed consent.
**Ethical approval:** The study was approved by Ethics Committee of Fuxing Hospital and was carried out in accordance with ethical guidelines of Ethics Committee of Fuxing Hospital.

## 3 Results

### 3.1 PCA analysis of samples from different groups

**Figure 1:**
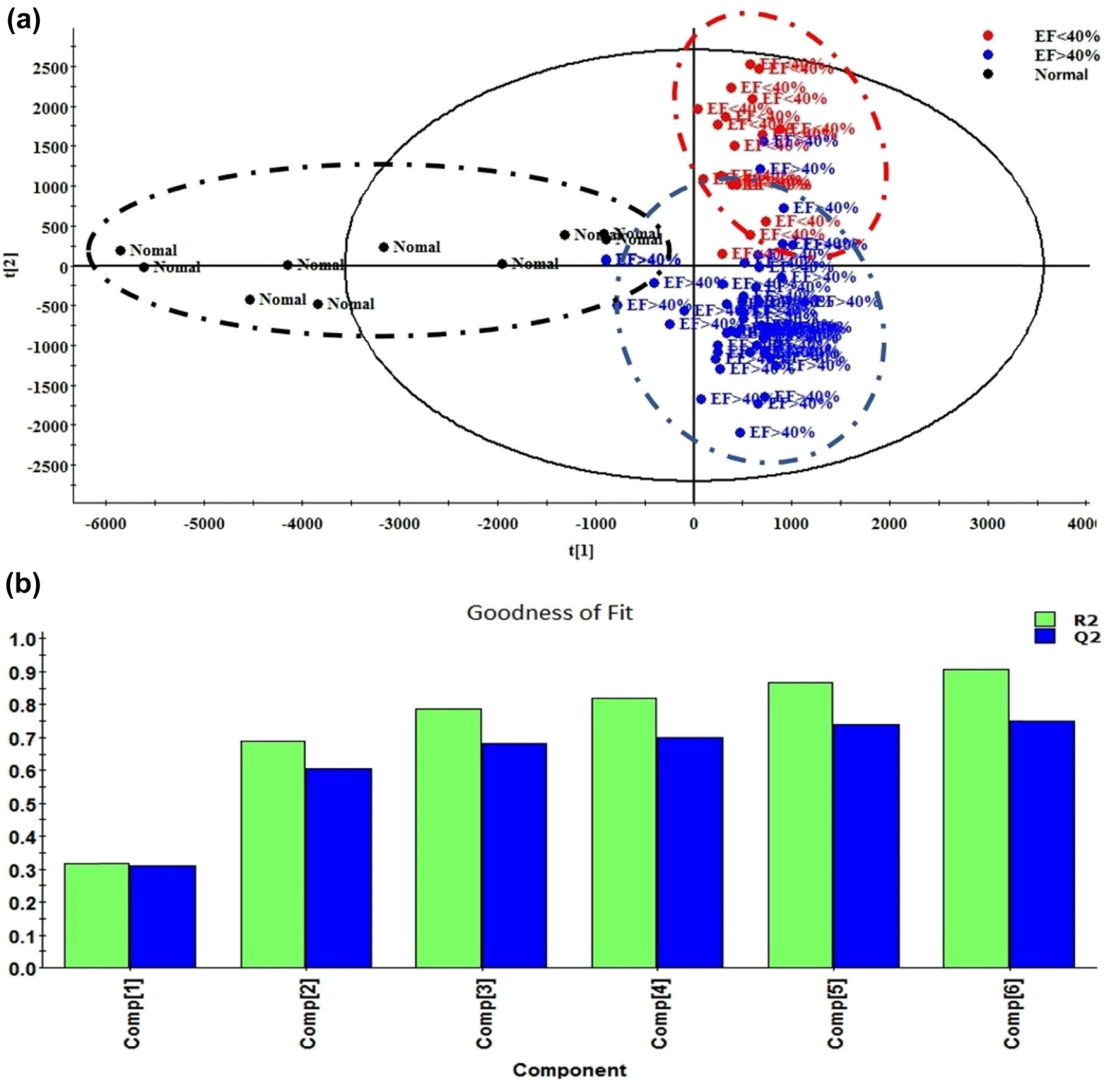
PCA of heart failure patient groups with different ejection fractions. (a) PCA chart, where black represents the group with preserved ejection fraction, blue represents the group of heart failure patients with an ejection fraction greater than 40%, and red represents the group of heart failure patients with an ejection fraction less than 40%. (b) Goodness of fit test of PCA diagram. Under the sixth principal component, the cumulative value of *R* ^2^ and *Q* ^2^ was close to one thousand, indicating good goodness of PCA analysis model.

As shown in Figure 1a, samples within the same group exhibited a good aggregation state, indicating good consistency in endogenous differential metabolites within the same group. There were significant differences in the contents of metabolites in different groups. The clustering circle of the group with normal ejection fraction (i.e., ejection fraction retention group) (EF Normal, EF ≥ 50%) was far from other groups, indicating significant difference in metabolites between the ejection fraction retention group and the group with mild reduction in ejection fraction (EF > 40%) and the group with reduced ejection fraction (EF < 40%). The difference in metabolites between patients with mild decrease in ejection fraction (EF > 40%) and those with reduced ejection fraction (EF < 40%) was relatively weak.

Figure 1b shows the fitting score of PCA model. Under the sixth principal component, the cumulative value of *R* ^2^ and *Q* ^2^ was close to one thousand, indicating good goodness of PCA analysis model.

### 3.2 Identification and analysis of differential metabolites in different groups

The differential metabolites were identified using fragment ions from relevant databases, mainly including lipid substances such as phosphatidylcholine (PC), phosphatidylinositol (PI), lysophosphatidylcholine (LysoPC), lysophosphatidylphosphatidylcholine (LysoPE), ceramide, sphingosine, and sphingomyelin (SM).

**Figure 2:**
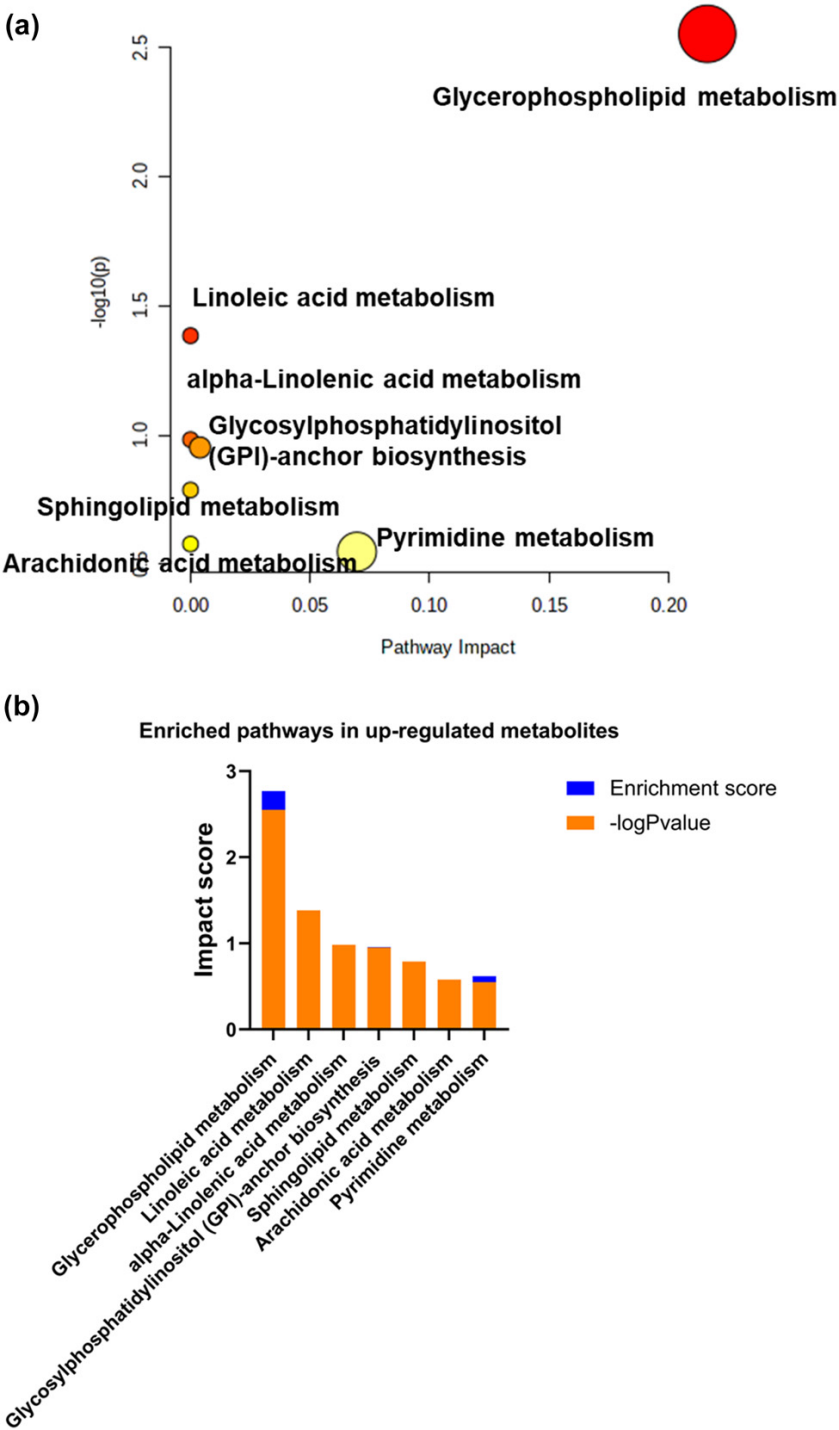
Metabolite enrichment analysis of three sets of differential metabolites. (a) Bubble diagram of metabolic enrichment. The larger the circle, the more important the metabolic pathways affected. (b) Importance value of metabolic pathway enrichment. The horizontal axis represented the important values of the metabolic pathway influence of differential metabolites, while the vertical axis represented the *p*-value size of the specialized metabolic pathway.

Enrichment of metabolic pathways was carried out on three groups of differential metabolites. As shown in Figure 2a, the horizontal axis represented the important values of the metabolic pathway influence of differential metabolites, while the vertical axis represented the *p*-value size of the specialized metabolic pathway. The darker the color of the enriched metabolic pathway, the smaller *p*-value; the larger the circle, the more important the metabolic pathways affected. Under these conditions, the glycerol phospholipid metabolism pathway, linoleic acid metabolism pathway, purine pyrimidine metabolism pathway, and linolenic acid metabolism were all significantly regulated (Figure 2b).

## 4 Discussion

HF is a serious and complex syndrome with high incidence rate and mortality, and no reliable indicators related to LVEF have been identified. Metabolomics is an innovative tool that can be used to detect new biomarkers of diseases [4,5]. Metabolomics has shown diagnostic value in evaluating metabolic disorders related to HF, and the distribution of metabolites provides better prognostic value compared to conventional biomarkers [6–8]. Therefore, metabolomics is expected to become a favorable tool for detecting biomarkers related to LVEF.

The liquid phase high-resolution mass spectrometry method used in this study has the characteristics of high sensitivity, high resolution, and good resolution, and has been widely used in basic and clinical metabolomics research in recent years. By using this method to detect the blood samples of patients with HFpEF, HFmrEF, and HFrEF, we identified seven potential biomarkers and several metabolic pathways related to the differences among HFpEF, HFmrEF, and HFrEF.

In this study, fragment ions from relevant databases were used to identify the differential metabolites. The differential metabolites in the three groups of patients with HFrEF, HFmrEF, and HFpEF mainly included lipid substances, such as PC, PI, LysoPC, LysoPE, ceramide, sphingosine, and SM. In terms of metabolic pathways, the glycerol phospholipid metabolic pathway, linoleic acid metabolic pathway, purine pyrimidine metabolic pathway, and linolenic acid metabolism were significantly regulated in the three groups of patients with HFrEF, HFmrEF, and HFpEF, with the glycerol phospholipid metabolic pathway being particularly important.

Glycerophospholipid metabolism is important for myocardial cells. LysoPCs, lysophosphatidylethanolamine (LysoPEs), lysophosphatidylserine, lysophosphatidic acid (LysoPEs), lysophosphatidylinositol, and lysophosphatidylglycerol can be produced after the hydrolysis of the side chain of glycerol phospholipid [9]. The metabolic abnormalities of lysophospholipids in the body are closely related to many diseases. As an important component of myocardial energy metabolism, glycerol phospholipids accelerate the degradation of myocardial cell membrane phospholipids when myocardial injury occurs, and may even be accompanied by severe mitochondrial function loss [10]. Cardiolipin (CL) is also synthesized during the metabolism of glycerol phospholipids. CL is one of the main phospholipids in inner mitochondrial membrane, which is related to the assembly and activity of various protein complexes in mitochondria. The respiratory chain complexes I to V and the proteins of the solute carrier family have shown tight binding with CL. CL not only binds to the surface of these proteins, but also promotes their assembly into supercomplexes and stabilizes their structure [11]. In HF, the loss of CL and tetralinolenic CL promotes the production of excessive reactive oxygen species, which are byproducts of inefficient mitochondrial electron transport chain complexes I and III, leading to cytochrome c catalyzed CL oxidation and myocardial cell apoptosis. Therefore, protecting CL and mitochondrial function may be key to preventing the development of HF [12].

Previous studies have shown close relationship between glycerophospholipid metabolism and HF. Miao et al. identified a total of 210 metabolites with significant differences between HF rats and normal rats. These metabolites mainly participate in tryptophan metabolism, branched chain amino acid metabolism, fatty acid metabolism, β oxidation, and glycerol phospholipid metabolism [13]. Wen et al. found that therapeutic effect of HF with hydrogenated amine combined with 6-gingerol was mainly related to the regulation of acetylphosphate, 3-carboxyl-1-hydroxypropylthiamine diphosphate, coenzyme A, palmitic acid, and oleic acid. Metabolic pathway analysis showed that the pathways affecting energy metabolism included glycerol phospholipid metabolism, fatty acid metabolism, pantothenic acid, coenzyme A biosynthesis, citric acid cycle (TCA cycle), pyruvate metabolism, and arachidonic acid metabolism [14]. The Linggui Zhugan Decoction could delay the pathological process of HF by regulating the disordered metabolic pathway and related enzymes, and the most crucial pathway is the metabolism of glycerol phospholipid and arachidonic acid [15]. However, none of the above studies have explored the metabolomic differences among HFrEF, HFmrEF, and HFpEF patients. In this study, blood samples from HFpEF, HFmrEF, and HFrEF patients were analyzed by metabonomics, and we identified seven potential biomarker and four metabolic pathways, but the underlying mechanisms still need to be further explored to develop novel biomarkers for chronic HF [16].

In conclusion, we analyzed blood metabonomics of 110 patients with congestive HF who were divided into three groups of HFpEF, HFmrEF, and HFrEF. The differential metabolites among them mainly include lipid substances, such as PC, PI, LysoPC, LysoPE, ceramide, sphingosine, and SM. The glycerol phospholipid metabolism pathway, linoleic acid metabolism pathway, purine pyrimidine metabolism pathway, and linolenic acid metabolism have all undergone significant regulation, among which the glycerol phospholipid metabolism pathway is particularly important. Changes in glycerol phospholipid metabolism pathway may help identify HFpEF, HFmrEF, and HFrEF.

## References

[1] Cantinotti M, Law Y, Vittorini S, Crocetti M, Marco M, Murzi B, et al. The potential and limitations of plasma BNP measurement in the diagnosis, prognosis, and management of children with heart failure due to congenital cardiac disease: an update. Heart Fail Rev. 2014;19(6):727–42. 10.1007/s10741-014-9422-2 24473828

[2] Wojtczak-Soska K, Sakowicz A, Pietrucha T, Lelonek M. Soluble ST2 protein in the short-term prognosis after hospitalisation in chronic systolic heart failure. Kardiol Pol. 2014;72:725–34. 10.5603/KP.a2014.0085 24846354

[3] Meng X, Liu Y, Xu S, Yang L, Yin R. Review on analytical technologies and applications in metabolomics. Biocell. 2024;48(1):65–78.

[4] Dong H, Yin C, Xiao D, Tang Y. Identification of differentially expressed genes to predict the risk of heart failure in older patients with hypertrophic cardiomyopathy. Aging (Albany NY). 2024;16(13):10860–7. 10.18632/aging.205956 PMC11272120 38972072

[5] Puetz A, Artati A, Adamski J, Schuett K, Romeo F, Stoehr R, et al. Non-targeted metabolomics identify polyamine metabolite acisoga as novel biomarker for reduced left ventricular function. ESC Heart Fail. 2022;9(1):564–73. 10.1002/ehf2.13713 PMC8788009 34811951

[6] Mofrad MD, Ahn S, Chun OK. The interplay of diet, genome, metabolome, and gut microbiome in cardiovascular disease: a narrative review. Curr Med Chem. 2025;32(30):6435–59.Epub ahead of print. 10.2174/0109298673342364241119114722 39779558

[7] Cheng ML, Wang CH, Shiao MS, Liu MH, Huang YY, Huang CY, et al. Metabolic disturbances identified in plasma are associated with outcomes in patients with heart failure: diagnostic and prognostic value of metabolomics. J Am Coll Cardiol. 2015;65(15):1509–20. 10.1016/j.jacc.2015.02.018 25881932

[8] Li Y, Hao C, Chen W, Meng Q. Analysis of specific lipid metabolites in cord blood of patients with gestational diabetes mellitus. Biocell. 2022;46(6):1565–73.

[9] Tsukahara T, Matsuda Y, Haniu H. Lysophospholipid-related diseases and PPARgamma signaling pathway. Int J Mol Sci. 2017;18(12):2730. 10.3390/ijms18122730 PMC5751331 29258184

[10] Law SH, Chan ML, Marathe GK, Parveen F, Chen CH, Ke LY. An updated review of lysophosphatidylcholine metabolism in human diseases. Int J Mol Sci. 2019;20(5):1149. 10.3390/ijms20051149 PMC6429061 30845751

[11] Schlame M, Greenberg ML. Biosynthesis, remodeling and turnover of mitochondrial cardiolipin. Biochim Biophys Acta Mol Cell Biol Lipids. 2017;1862(1):3–7. 10.1016/j.bbalip.2016.08.010 PMC5125896 27556952

[12] Dolinsky VW, Cole LK, Sparagna GC, Hatch GM. Cardiac mitochondrial energy metabolism in heart failure: role of cardiolipin and sirtuins. Biochim Biophys Acta. 2016;1861(10):1544–54. 10.1016/j.bbalip.2016.03.008 26972373

[13] Miao X, Chen J, Su Y, Luo J, He Y, Ma J, et al. Plasma metabolomic analysis reveals the therapeutic effects of Jiashen tablets on heart failure. Front Cardiovasc Med. 2022;9:1047322. 10.3389/fcvm.2022.1047322 PMC9763324 36561767

[14] Wen J, Ma X, Niu M, Hao J, Huang Y, Wang R, et al. Metabolomics coupled with integrated approaches reveal the therapeutic effects of higenamine combined with [6]-gingerol on doxorubicin-induced chronic heart failure in rats. Chin Med. 2020;15(1):120. 10.1186/s13020-020-00403-0 PMC7670783 33292391

[15] Wang X, Gao Y, Tian Y, Liu X, Zhang G, Wang Q, et al. Integrative serum metabolomics and network analysis on mechanisms exploration of Ling-Gui-Zhu-Gan Decoction on doxorubicin-induced heart failure mice. J Ethnopharmacol. 2020;250:112397. 10.1016/j.jep.2019.112397 31830550

[16] Wang W, Wu Y, Zhang Q, Cui P. Metabolome profiling of malignant ascites identifies candidate metabolic biomarkers of hepatocellular carcinoma. Curr Med Chem. 2024;31(13):1769–80. 10.2174/0929867330666230324153552 38666505

